# Dynamics of Leaf-Litter Biomass, Nutrient Resorption Efficiency and Decomposition in a Moso Bamboo Forest After Strip Clearcutting

**DOI:** 10.3389/fpls.2021.799424

**Published:** 2022-01-27

**Authors:** Yaxiong Zheng, Fengying Guan, Shaohui Fan, Xinrong Yan, Lanying Huang

**Affiliations:** ^1^Key Laboratory of National Forestry and Grassland Administration, International Center for Bamboo and Rattan, Beijing, China; ^2^National Location Observation and Research Station of the Bamboo Forest Ecosystem in Yixing, National Forestry and Grassland Administration, Beijing, China

**Keywords:** moso bamboo forest, strip clearcutting, litterfall, resorption efficiency, nutrient return

## Abstract

Strip clearcutting can significantly reduce the harvesting costs of moso bamboo forests. Although bamboo is characterized by rapid accumulation of biomass, it is still a concern that this management method may reduce long-term productivity. Nutrient cycling has long been considered essential for forests to maintain high primary productivity. However, nutrient cycling of bamboo forests after strip cutting has not been previously reported. We conducted a strip clearcutting experiment and surveyed the litter dynamics for 1 year. We assessed changes in litter nutrients in response to the cutting and calculated the nutrient resorption efficiency and litter decomposition rate to evaluate the effect on nutrient use efficiency and nutrient return. Our results showed that strip cutting had no significant effect on litter production and nutrient return in the moso bamboo forest (*p* > 0.05). However, annual litter biomass and nutrient return in reserved belts (RB) were significantly higher than those in the control (CK) (*p* < 0.05). P and K resorption efficiencies in RB were significantly higher than in CK during certain periods of bamboo growth (*p* < 0.05). We also observed that the annual decay constant of CK was significantly higher than that of plots that were strip clearcut (SC) (*p* < 0.05). Our results suggest that strip cutting does not affect nutrient use efficiency or storage in the short term.

## Introduction

Bamboo is an important forest type in China (Su et al., 2019a). More than 500 species of bamboo in 39 genera are spread across 13 provinces (Jiang, 2007). Moso bamboo (*Phyllostachys edulis* (Carrière) J. Houz.) covers 4.67 million ha (72.96%) of the total bamboo forest area in China (Jiang, 2007). Moso bamboo trees can be harvested after 4 years and have gradually become a substitute for wood (Su et al., 2019a), being widely adopted as construction material, furniture, biomass energy, and other related uses (Jiang, 2007). Bamboo shoots are also a tasty and nutritious food (Jiang, 2007). Therefore, moso bamboo plays an important role in the development of the forest economy in China (Zhao et al., 2018a).

Moso bamboo is characterized by clonal integration and is a typical forest comprising plants of different ages (Su et al., 2019a). In traditional harvesting, trees older than 4 years require their age to be identified by an experienced farmer (Zhao et al., 2018b). However, due to development of the social economy and population urbanization, the rising costs caused by labor shortages have reduced the enthusiasm of bamboo farmers. Fortunately, the demand to reduce the management costs of bamboo forests has been effectively supported. The strip-cutting model has been studied in a major region of moso bamboo forest as a possible solution to reduce the high cutting cost (Li et al., 2018; Zeng et al., 2019b; Zhan et al., 2020). Felled bamboo is taken out of the cutting site for sale. Previous studies have analyzed the natural restoration status of postharvest plots with different widths, and suggested that 8 m is the best width for strip cutting (Li et al., 2018; Xianli, 2019; Zhan, 2019). However, bamboo forest development mainly relies on the recycling of nutrients from soil organic matter to sustain fertility. Rotation is a common management practice. Therefore, there are some concerns that these practices may reduce long-term productivity (Turner and Lambert, 2013).

Litterfall is the main bridge between vegetation and mineral soil and plays a central role in the nutrient cycle of forest ecosystems (Huang et al., 2017; Johnson and Turner, 2019). By assessing differences in litterfall yield and chemical properties, we can gain important insights into the impact of logging on the ecological function of moso bamboo communities (Zhou et al., 2007). Litter, through nutrient deposition, has been reported to provide more than 70% of plant growth nutrients (Adolfo et al., 2016). Litter substrate (Lihua et al., 2014) and environmental conditions (Bothwell et al., 2014) determine the decomposition rate of organic matter return. Nutrient resorption refers to the transport of movable proteins, carbohydrates, and other nutrients from aging tissues and organs to other tissues to ensure that they remain in the plant and are available for physiological processes and future growth demands (Tang et al., 2013). The nutrient content of leaves is closely related to soil nutrition and bamboo yield in the following year (Chen et al., 2009). When the availability of soil nutrients decreases, the internal reabsorption level is higher, indicating that nutrient concentration in the litter also decreases (Johnson and Turner, 2019). Research on the dynamics of nutrient resorption in leaves will improve our understanding of nutrient retention, utilization, and adaptation to the development environment of plants (Proe and Millard, 1995). At the stand level, a change in nutrient resorption is reflected by the nutrient concentration and mass of litter (Turner and Lambert, 2015). This is of great interest, as it is directly related to the nutrient return of the stand.

We have studied the effects of strip cutting on bamboo restoration characteristics (Su et al., 2019b; Zeng et al., 2019a), undergrowth vegetation diversity (Zhan et al., 2020), and soil nutrient characteristics (Zeng et al., 2019b; Zhan, 2019). However, the effect of strip cutting on litterfall and the nutrient cycling of moso bamboo forests is unclear. This may limit our ability to predict the long-term productivity of bamboo forests. Hence, it is necessary to gain a wider understanding of how cutting affects litter conditions (e.g., productivity and related nutrients) to guide the restoration and realization of healthy and sustainable management of cut bamboo forests. In this study, we quantified litterfall and nutrient return, determined the nutrient resorption efficiency as well as the rates of litter decomposition of different treatments of moso bamboo to determine the patterns of nutrient release on the floor by thinning practices. We tested the hypotheses that (1) strip cutting has no significant effect on nutrient return; (2) cutting reduces the density of bamboo forests and provides a sufficient nutrient environment for the growth of stand development; and (3) cutting may affect the litter decomposition rate. This study was conducted on experimental bamboo plantations in subtropical China.

## Materials and Methods

### Study Site

Our study was performed in the Yixing Forest Farm (31°15′1″-31°15′12″ N, 119°44′2″-119°44′8″ E), located in southern Jiangsu Province, China. This region is dominated by low mountainous and hilly terrain, and the soil type is identified as yellow clay soil according to the classification and codes for Chinese soil (GB/T 17296-2009). The area is characterized by a maritime monsoon climate. The lowest and highest annual temperatures are −4.5°C and 38.8°C, respectively, with an annual average temperature of 16.5 °C (data from Yixing Forest meteorological station, located 1 km from the field station). There are 129 rainy days per year, with an average annual precipitation of 1229.9 mm. The growing period can reach approximately 250 days. The farm was established in March 1950 with 120 ha of monodominant moso bamboo forest. There are no pests, diseases, or fertilization treatments in the region. The average bamboo DBH and height is 8.13 cm and 13.37 m, respectively, and the average tree density is 3375 stems ha^–1^. The on-year and off-year can be clearly distinguished. The prominent understory species include *Hedyotis chrysotricha*, *Carex breviculmis*, *Paederia cruddasiana*, *Oxalis corniculata*, and *Salvia prionitis*.

**FIGURE 1:**
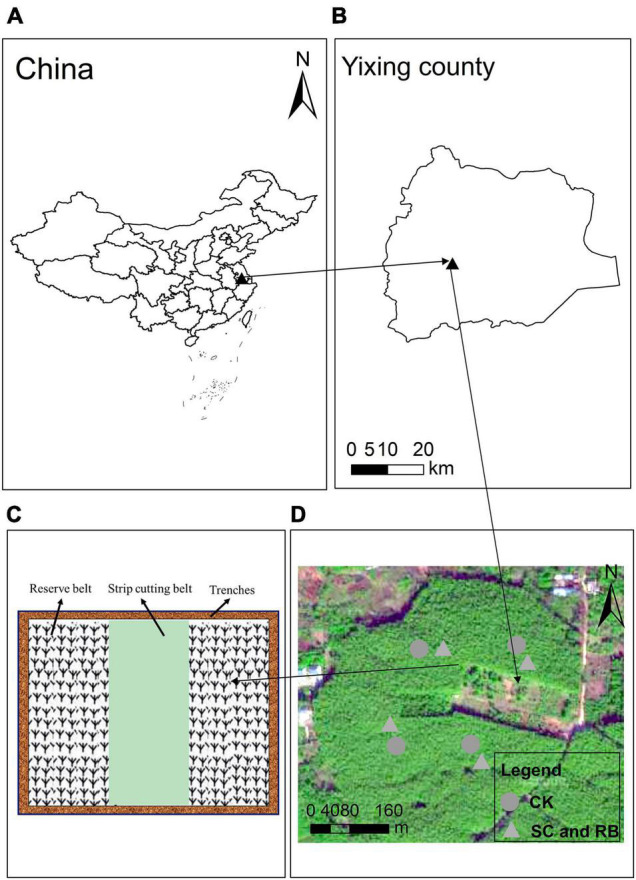
Location of the study area **(A,B)** and field experiment design **(C,D)** and sampling sites (SC, RB, and CK) Moso bamboo forests.

Strip clearcutting involves cutting all trees in the plot and removing them from the experimental site. Two reserved belts were set between each cut belt. The function of the reserved plots was to provide nutrients to the strip-cut belts through physiological integration. And we need to know that physiological integration is one of the important characteristics of clonal plants. When the linked clonal ramet is located in different resource patches, the material absorbed from the resource-rich patch can be shared by other connected ramets outside the patch, that is, the ability to resist disturbance is improved (Su et al., 2019a). No management practices were carried out in the harvest plots and reserve plots during the restoration period. The test was performed at the same site quality. This study designed three different types of sites as treatments, including unharvested plots as a control (CK), plots that were strip clearcut (SC) in February 2019, and the reserved belt (RB). The cutting plots were 24 m × 20 m, including a strip-cutting belt 8 m wide and 20 m long, and two reserved plots of the same size. Each treatment included four replicates. Moreover, four trenches 50 cm wide and 50 cm deep were excavated around the plot to cut off rhizomes, eliminating the effect of long-distance nutrient transport. A control treatment with (8 m × 20 m) uncut plots (CK) was also included. The slope was approximately 6°. The location of the study area and field experiment design and sampling sites was shown in Figure 1.

### Litterfall Measurements

Litterfall was measured between October 2019 and September 2020. Three litter traps (1 m × 1 m) at a height of 1 m were randomly placed along the median line in the SC, RB, and CK plots. The litter was collected monthly and dried at 65°C to a constant weight. An electronic balance (0.01 g) was used to weigh the samples. The samples were then ground into a fine powder, passed through a sieve with a diameter of 0.15 mm, and stored in a sealing bag to analyze the nutrient content.

### Mature Leaves Collection

Fresh leaves were collected from two stands in July 2019. Samples were taken according to the upper, middle, and lower layers along the tree heights. Ten bamboo trees were selected from each SC plot, and 20 bamboo trees were selected from four age grades in the CK plot. The leaves collected from the same plot were mixed into one composite sample. According to the physiological characteristics of moso bamboo, a 1-year-old bamboo was labeled as “du,” and thereafter marked as a sub-degree bamboo according to a 2-year vegetative cycle (Tang et al., 2016). For example, 2- to 3-year-old bamboos are labeled II “du,” and III “du” represented a 4- to 5-year-old bamboo forest. Therefore, we sampled new leaves in July 2020. The nutrient content of mature leaves from October 2019 to June 2020 was determined using the July 2019 survey. Mature leaves from July to September 2020 were represented by samples from July 2020. Freshly fallen litter and fresh leaves were dried at 65°C to a constant weight. The samples were then ground into a fine powder and passed through a sieve with a diameter of 0.15 mm, and the nutrient content was determined.

### Litter Decomposition

Freshly fallen litter was collected on nylon mesh screens in all stands from July to September 2019. We then used this to create litter decomposition bags. Part of the litter was collected, ground into a fine powder, and passed through a sieve with a diameter of 0.15 mm. The chemical properties were measured as the initial values of the litter bags. Litter decomposition bags (20 cm × 30 cm in size) were made of 1 mm mesh nylon net. Next, 20.00 g of dried litter was placed in each bag to measure the initial weight. In August 2019, we randomly placed bags in each plot. The bag was attached to the soil and fixed using a PVC pipe. Three litter bags were collected from each plot at 3-month intervals for 1 year. The recycled bag was washed with clean water to remove excess soil, dried at 65 °C to a constant weight, and then weighed. The samples were then ground into a fine powder, passed through a sieve with a diameter of 0.15 mm, and stored in a sealing bag to analyze the nutrient content.

### Soil Sampling

In each plot, sequential soil coring was used to extract 10 soil cores in October 2020. Sampling was conducted at a depth of 0–10 cm. Cores from the same layer were mixed into a composite sample. Coarse roots were removed from the mixed samples through a 2 cm sieve, and the soil chemical properties were determined after air-drying.

### Chemical Analysis

The soil organic carbon (SOC) and total nitrogen (TN) content was determined using an elemental analyzer (ECS 4024 CHNSO; Costech, Picarro, Italy). Total phosphorus (TP) content was determined following the molybdenum-antimony resistance colorimetric method (concentrated H_2_SO_4_-HClO_4_) using an automatic chemical analyzer (Smartchem 300; AMS, Italy). Total potassium (TK) content was determined using a flame photometer (M410; Sherwood, United Kingdom).

### Calculations and Statistics

Nutrient return was calculated as the product of litter mass and litter nutrient concentrations (Sayer et al., 2020). The nutrient resorption efficiency of plant leaves was calculated using the following formula (Guo et al., 2021):


(1) $$\text{Ri}=\frac{C_{0}-C_{t}}{C_{0}}\times 100\%$$

The Olson litter decomposition index model was adopted to calculate the decomposition rate (Olson, 1963):


(2) $$\frac{M_{t}}{M_{o}}=ae^{-kt}$$

(3) $$t_{0.5}=-\ln\left(0.5\right)/k$$

(4) $$t_{0.95}=-\ln\left(0.05\right)/k$$

The element remaining (ER) in the litter bag for each period (X_i_) was determined using the following formula (Ren et al., 2018):


(5) $$\text{ER}\left(\%\right)=\frac{\left(M_{t}\times C_{t}\right)}{\left(M_{0}\times C_{0}\right)}\times 100\%$$

where, Ri (%) denotes the resorption efficiency of nutrient i (N,P,K) in percent, C_0_ is the nutrient content of mature leaves, C_t_ is the nutrient content of litters at time t, M_0_ is the initial dry mass of litter, M_t_ is the remaining dry mass of litter decomposed at time t (in years), k is the annual decay constant, t_0.5_ is the time for 50% of mass loss, and t_0.95_ is the time for 95% of mass loss.

One-way analysis of variance (ANOVA) was used to test the differences between the three treatment plots. Means were separated by the least significant difference (LSD) test, and statistical significance was set at *p* < 0.05. Principal component analysis (PCA) was used to examine the associations between annual litter yield, nutrient return, and soil characteristics. All statistical analyses were performed in R (version 3.6.2), and the data calculated using Excel 2016. We examined the assumptions of normality and homogeneous variance using the Shapiro-Wilk test and Leven’s test, respectively. PCA was calculated using the FaceFactoMineR package. All graphs were drawn using the ggplot2 package.

## Results

### Litterfall and Nutrient Return

The trends of monthly litter in the three sites were the same, with two increase-decrease cycles. As shown in Table 1, the litter biomass from March and April 2020 in RB was significantly higher than in SC and CK (*p* < 0.05). From July to September 2020, the litter yield of SC was significantly lower than that of CK and RB (*p* < 0.05). We also found that the annual litter yield of RB was significantly higher than that of CK and SC (*p* < 0.05).

**TABLE 1:** Monthly and annual litter yield of strip clearcut (SC), reserve belt (RB), and unharvested (CK) plots.

Stands	October 2019 (g m^–2^)	November 2019 (g m^–2^)	December 2019 (g m^–2^)	January 2020 (g m^–2^)	February 2020 (g m^–2^)	March 2020 (g m^–2^)	April 2020 (g m^–2^)	May 2020 (g m^–2^)	June 2020 (g m^–2^)	July 2020 (g m^–2^)	August 2020 (g m^–2^)	September 2020 (g m^–2^)	Mass sum (g m^2^ year^–1^)
SC	10.70 ± 1.72a	42.61 ± 2.47a	10.26 ± 1.81a	9.37 ± 1.05a	7.20 ± 1.02a	51.28 ± 14.65a	156.23 ± 13.18a	117.18 ± 16.17a	24.14 ± 2.98a	7.75 ± 1.45a	4.02 ± 0.90a	2.11 ± 0.46a	442.83 ± 18.59a
RB	19.15 ± 2.14b	53.87 ± 6.22b	14.17 ± 1.55b	11.84 ± 0.99b	7.99 ± 1.94a	73.28 ± 6.48b	199.65 ± 36.24b	99.12 ± 15.03ab	27.15 ± 4.32a	11.97 ± 1.68b	5.87 ± 0.90b	2.82 ± 0.54b	526.89 ± 50.01b
CK	16.27 ± 2.22b	47.86 ± 7.97ab	11.92 ± 1.95ab	10.41 ± 1.50ab	8.31 ± 1.15a	52.69 ± 11.98a	135.79 ± 26.07a	86.23 ± 12.44b	25.53 ± 5.36a	14.37 ± 2.39b	6.35 ± 0.63b	3.12 ± 0.22b	418.86 ± 69.38a

*Values are the mean ± standard deviation (n = 4). Different lowercase letters within a column indicate a significant difference in litter yield between the three types of plots (ANOVA and LSD test, p < 0.05).*

**FIGURE 2:**
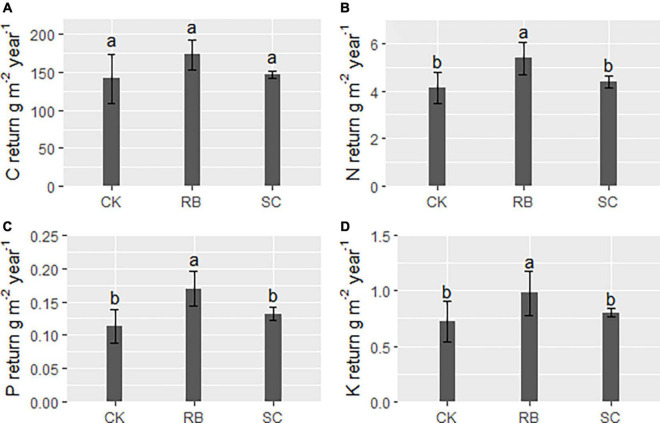
Return of elements (**A**, carbon; **B**, nitrogen; **C**, phosphorus; and **D**, Potassium) via litter to forest soil in strip clearcut (SC), reserve belt (RB), and unharvested (CK) plots within a year. Different lowercase letters indicate a significant difference in element return between the three type plots (ANOVA and LSD test, *P* < 0.05). Error bars indicate standard deviation (*n* = 4).

The annual return amounts of N, P, and K in RB were significantly higher than those in SC and CK (Figure 2, *p* < 0.05). However, there was no significant difference in C return between the three plot types (Figure 2, *p* > 0.05).

### Nutrient Resorption Efficiency

From September 2019 to August 2020, the N, P, and K resorption efficiencies in the three types of plots showed two increase-decrease cycles. For all treatments, N resorption efficiency was the highest in May 2020. In May and August 2020, N resorption efficiency in SC was significantly lower than in the CK (Table 2). For all treatments, P resorption efficiency was the highest in April 2020, where there was no significant difference between the treatments. However, P resorption efficiency in RB was significantly higher than in CK in February and June 2020. For all treatments, K resorption efficiency was the highest in December 2019, where there was no difference between the three treatments. In January and April 2020, K resorption efficiency in SC was significantly lower than in CK. From July to September 2020, K resorption efficiency in SC was significantly higher than in the CK. In June and July 2020, the K reabsorption efficiency of RB was significantly higher than that of CK (Table 2).

**TABLE 2:** Nutrient resorption efficiency of moso bamboo in strip clearcut (SC), reserve belt (RB), and unharvested (CK) plots.

Time	Treatment	RN	RP	RK
		**(%)**	**(%)**	**(%)**
October 2019	SC	50.90 ± 2.96a	70.86 ± 3.83a	71.74 ± 1.72a
	RB	50.03 ± 3.45a	72.85 ± 5.60a	79.07 ± 5.24b
	CK	53.25 ± 1.77a	68.73 ± 11.86a	77.07 ± 2.74ab
November 2019	SC	56.53 ± 2.64a	77.29 ± 3.12a	92.59 ± 2.66a
	RB	59.37 ± 2.35a	76.39 ± 2.72a	92.90 ± 2.14a
	CK	59.30 ± 1.98a	74.79 ± 4.89a	92.34 ± 0.84a
December 2019	SC	59.64 ± 2.44a	75.70 ± 6.05a	93.47 ± 0.90a
	RB	66.50 ± 5.26b	78.64 ± 1.78a	96.09 ± 1.45a
	CK	60.57 ± 2.89ab	78.86 ± 2.37a	94.75 ± 2.44a
January 2020	SC	59.69 ± 1.58a	74.98 ± 7.42a	93.03 ± 0.07a
	RB	57.21 ± 2.53a	74.85 ± 4.31a	93.96 ± 0.83ab
	CK	60.65 ± 3.48a	75.03 ± 4.62a	94.34 ± 1.01b
February 2020	SC	55.17 ± 0.88a	65.26 ± 3.01a	93.04 ± 0.06a
	RB	55.10 ± 2.47a	73.89 ± 1.88b	93.62 ± 0.92a
	CK	56.50 ± 1.28a	68.75 ± 2.50a	92.76 ± 1.01a
March 2020	SC	62.96 ± 1.79a	77.75 ± 4.53a	90.44 ± 0.97a
	RB	61.16 ± 0.97a	79.00 ± 4.05a	92.55 ± 0.85b
	CK	61.77 ± 2.57a	75.72 ± 3.77a	92.34 ± 1.67ab
April 2020	SC	66.13 ± 1.78a	79.68 ± 2.18a	80.87 ± 1.46a
	RB	64.17 ± 1.20a	81.19 ± 1.58a	84.76 ± 1.56b
	CK	66.41 ± 1.67a	80.33 ± 2.64a	84.33 ± 1.42b
May 2020	SC	68.40 ± 0.81a	77.50 ± 1.66a	80.44 ± 1.60a
	RB	69.92 ± 0.89b	80.71 ± 2.62a	80.49 ± 3.53a
	CK	70.30 ± 1.08b	78.95 ± 3.98a	80.67 ± 5.09a
June 2020	SC	61.16 ± 1.79a	77.57 ± 2.63ab	90.46 ± 1.71ab
	RB	61.12 ± 2.18a	79.65 ± 1.13a	93.25 ± 1.50a
	CK	61.19 ± 2.15a	74.20 ± 2.44b	89.55 ± 3.08b
July 2020	SC	50.48 ± 1.50a	73.66 ± 2.22a	78.77 ± 4.74a
	RB	47.13 ± 2.46a	68.63 ± 5.30a	71.23 ± 4.93a
	CK	49.15 ± 4.45a	70.74 ± 3.99a	57.49 ± 6.61b
August 2020	SC	37.40 ± 3.71a	59.43 ± 4.69a	60.69 ± 1.91a
	RB	37.94 ± 4.82ab	58.19 ± 4.98a	51.09 ± 7.70b
	CK	45.62 ± 6.45b	61.91 ± 4.43a	44.49 ± 0.27b
September 2020	SC	39.81 ± 3.97a	61.05 ± 14.06a	65.34 ± 8.54a
	RB	43.02 ± 5.43a	65.94 ± 4.69a	53.14 ± 12.78b
	CK	40.04 ± 3.73a	61.60 ± 2.12a	35.63 ± 8.42b

*Values are the mean ± standard deviation (n = 4). Different lowercase letters within a column indicate a significant difference in element resorption efficiency between the three types of plots (ANOVA and LSD test, p < 0.05). RN, resorption efficiency of nitrogen; RP, resorption efficiency of phosphorus; RK, resorption efficiency of potassium.*

### Litter Decomposition

The k value of CK was significantly higher than that of the SC (Table 3, *p* < 0.05). After decomposing for 1 year, the residual rate of the dry mass of litter in CK, SC, and RB showed no significant difference (*p* > 0.05). The decomposition time of 50 and 95% mass loss of the litter in CK was significantly lower than that in SC (*p* < 0.05).

**TABLE 3:** Decomposition parameters of litter in strip clearcut (SC), reserve belt (RB), and unharvested (CK) plots.

Treatment	Equation	*R* ^2^	Remaining rate (%)	k (year^–1^)	t_0.5_ (year)	t_0.95_ (year)
SC	y = 97.887e^–0.288t^	0.961	72.63 ± 1.40a	0.29 ± 0.01a	2.41 ± 0.04a	10.41 ± 0.18a
RB	y = 97.912e^–0.299t^	0.961	72.18 ± 1.20a	0.30 ± 0.01ab	2.33 ± 0.10ab	10.05 ± 0.45ab
CK	y = 102.21e^–0.316t^	0.963	73.05 ± 0.20a	0.32 ± 0.01b	2.20 ± 0.08b	9.50 ± 0.35b

*Values are the mean ± standard deviation (n = 4). Different lowercase letters within a column indicate significant differences between treatments (ANOVA and LSD test, P < 0.05). k, annual decay constant; T_0.5_, time for 50% mass loss; T_0.95_, time for 95% mass loss.*

### Bioelement Dynamics

**FIGURE 3:**
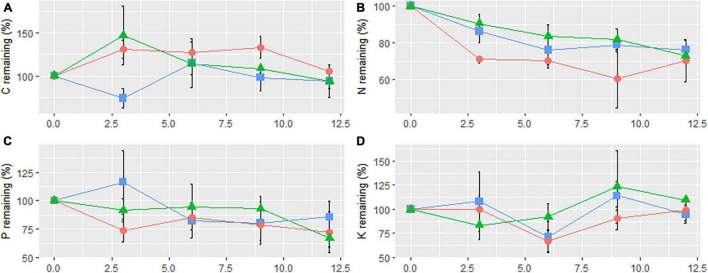
Elements remaining of carbon **(A)**, nitrogen **(B)**, phosphorus **(C)**, and potassium **(D)** of litter bag on strip clearcut (SC, rectangle), reserve belt (RB, triangle), and unharvested (CK, circle) plots. The error bars indicate the standard deviation (*n* = 4).

The concentration dynamics of the remaining four bioelements exhibited various patterns during the year-long decomposition process in the three treatment plots (Figure 3). The remaining concentration of C in CK exhibited two increase-decrease cycles, and the final concentration had increased (Figure 3A). C concentration decreased in the initial stage (decrease-increase-decrease) in SC, but increased in the initial stage (increase-decrease) in RB. The final C concentrations in both sites decreased. N concentration in the three types of plots decreased in the initial stage, but the concentrations fluctuated upward in CK and SC during the decomposition process (Figure 3B). In general, litter from all types of sample sites released a partial amount (>20%) of N during the decomposition process in 1 year. P concentration in CK and RB decreased in the initial stage (decrease-increase-decrease) (Figure 3C). In contrast, P concentration increased in the initial stage (increase-decrease-increase) in SC. The final P concentration decreased in all plots. K concentrations in SC, RB, and CK remained consistent with initial content (Figure 3D).

## Discussion

### Effect of Strip Cutting on Litterfall and Nutrient Return

In subtropical bamboo ecosystems with limited nutrition, strip cutting can effectively reduce harvesting costs. Moso bamboo is a fast-growing grass, and the nutrients needed in the different stages of bamboo growth are mainly provided by the physiological integration of the trees in the RB (Su et al., 2019a), but the development of stands relying only on the soil nutrient cycle maintains fertility. The production and nutrient content of litter plays a key role in the soil nutrient pool (Lihua et al., 2014). Litterfall is used as an indicator of functional recovery to assess tropical cloud forest restoration (Guadalupe et al., 2021). Our study found that annual litterfall did not differ significantly between SC and CK, but increased significantly in RB. This result does not coincide with other types of forests, such as *Cunninghamia lanceolata* (Jian et al., 2021), poplars (Berthelot et al., 2000), and the Sari Bumi Kusuma forest (Prasetyo et al., 2015). There are numerous possible mechanisms involved in litterfall production. First, the litter yield was closely related to the density of bamboo, and the number of bamboo trees in SC was significantly reduced (Zeng et al., 2019a). Second, the age of the bamboo trees also affected litter yield. New trees change leaves after 1 year of growth, whereas trees older than 1 year change leaves every 2 years (Su, 2012). Moreover, the allocation of biomass in the cutting plots was also the main factor affecting litter return. Bamboo in strip cutting plots may increase photosynthesis by increasing leaf biomass, which can provide more nutrients for the development of the rhizome system. In addition, we cannot eliminate the possibility that litters from the RB fell into the strip cutting plot due to wind.

In this study, there was no difference in the return amount of different nutrient elements between SC and CK, which indicates that strip cutting does not decrease nutrient return. This finding is consistent with our hypothesis. However, the return amounts of N, P, and K in RB were significantly higher than those in CK and SC. This correlated with changes in litter yield (Figure 3). Conversely, there was no difference in C return between the three treatments. This difference may be due to the low nutrient content in the leaves of old bamboo. Guo (2014) tested nutrient content in the leaves of 1- to 6-year-old bamboo trees and found that C content in bamboo leaves decreased gradually with age, and that N and P content in bamboo leaves indicated a “W” shaped change with age.

### Effect of Strip Cutting on Nutrient Resorption Efficiency

According to the theory of dynamic equilibrium in ecological stoichiometry, when the external environment fluctuates within a certain narrow range, a steady-state mechanism can be formed in the organism to maintain stable nutrient elements and achieve dynamic equilibrium (Zhang et al., 2003). Strip cutting removed all aboveground biomass from the plot and altered the forest microenvironment. Nutrient resorption provides nutrients to developing tissue instead of the soil (Johnson and Turner, 2019). Nutrients in the leaves can be resorbed in plants through resorption and redistribution to adapt to a low supply of nutrients in the soil (Regina et al., 2000). Su (2012) studied the different growth stages of moso bamboo and found that N consumption was higher in the mature stage and vigorous vegetative growth stage, whereas the demand for P and K was higher in the whip stage of pregnant bamboo shoots. Our results indicated a decrease in N, P, and K resorption efficiency from June to September in 2020, which may indicate lower retranslocation of this element from the leaves to other organs during leaf renewal.

Nitrogen is essential for achieving good yields in a moso bamboo forest, because the demand for and uptake of N is the highest in each growing period (Su, 2012). In the three treatment plots, the resorption efficiency of N tended to increase during the growth cycle (Table 2). This enhances the use efficiency of N, which might maintain balance in the internal supply of nutrients between the soil and the plant, with trees taking advantage of the most accessible routes (Miller et al., 1979; Turner and Lambert, 2015). The nutrient reabsorption capacity of N was strong, indicating that the availability of soil N was weak. Under N stress, moso bamboo can increase nutrient resorption efficiency and store more nutrients in the old leaves when the nutrient supply is sufficient. We found that N resorption efficiency in CK was significantly higher than in the SC in May and August 2020, which could reduce the limit of N, possibly owing to the high density of bamboo in CK. Phosphorus is an essential micronutrient for higher plants and is usually a highly mobile and frequently translocated element (White, 2012). Our study found that there was no difference in the reabsorption efficiency of P between SC and CK, but P resorption efficiency in RB was significantly higher than in CK in February and June 2020 (Table 2). Previous reports have also discussed different trends in seasonal variation, showing that P translocates from the leaves to other organs (Umemura and Takenaka, 2014). Potassium accumulates in meristems and young tissues, and is assimilated to the roots of higher plants (White, 2012). Umemura and Takenaka (2014) investigated K loss from the leaves and observed K resorption from mature leaves to growing organs. In our study, K showed the highest resorption efficiency (Table 2), due to the retranslocation of K. Additionally, the leaching of rainfall also has a great influence on K loss. Sakai and Tadaki (1997) studied the K^+^ concentration of forest rainfall in *Phyllostachys pubescens* and verified that K^+^ had leached from bamboo leaves.

### Effect of Strip Cutting on Litter Decomposition and Bioelement Dynamics

Litter decomposition is a basic biogeochemical cycle in forest ecosystems (Lihua et al., 2014). There are a number of factors that affect the litter decomposition rate, including temperature (Bothwell et al., 2014), precipitation (Wieder et al., 2009), litter substrate quality (Astel et al., 2009), and soil nutrient availability (Keeler et al., 2009). In the present study, we found that litter decomposition rates were significantly higher in CK than in SC (Table 3). There was a slower release of nutrients from litter decomposition in SC, which contributed to the storage of soil nutrients. Moso bamboo is a fast-growing plant. It requires a high nutrient cycling rate in high-density stands. The decrease in nutrient deposition in the postharvest plots may be influenced by bamboo density. A previous study suggested that, in a higher fertility soil, plants tend to obtain nutrients from the soil more efficiently, rather than to accelerate litter decomposition and absorption (Wright and Westoby, 2010). We suggest that the reduction in bamboo density has created a more nutrient-rich environment in postharvest sites. In addition, the effect of the functional diversity of soil fauna on the decomposition rate over a short period of time should also be considered (Bohlen et al., 2004). Luan et al. (2020) found that the expansion of moso bamboo slowed down the decomposition rate of litter, but this negative effect was reversed when the role of macrofauna was excluded. Forest clearing significantly decreased the species richness of soil macrofauna and altered the composition (Mathieu et al., 2010). Therefore, strip cutting may change soil fauna diversity and composition, which results in a lower litter decomposition rate in SC.

After 1 year, the three treatment plots indicated a net release of N and P, whereas C and K release had an opposite trend (Figure 3). Parton et al. (2007) found that only the average C/N ratio of the litter was less than 40, indicating that net N release had occurred. A previous study, focused on nutrient dynamics in *Pleioblastus amarus* and *Bambusa pervariabilis* × *Dendrocalamopsis daii* stands, found that the trophic dynamic pattern and the remaining final quantities were mainly determined by their initial matrix quality (Lihua et al., 2014). This observation is consistent with our findings. The pattern of bioelement dynamics and the elements remaining in RB and CK changed consistently (Figure 3). However, in SC, the change differed. After decomposing for 3 months, nutrient release in SC indicated a net C release and net P enrichment. This may be due to the demands of decomposers and the availability of nutrients in the environment (Lihua et al., 2014).

### Associations Between Annual Litter Yield and Nutrient Return and Soil Characteristics

**FIGURE 4:**
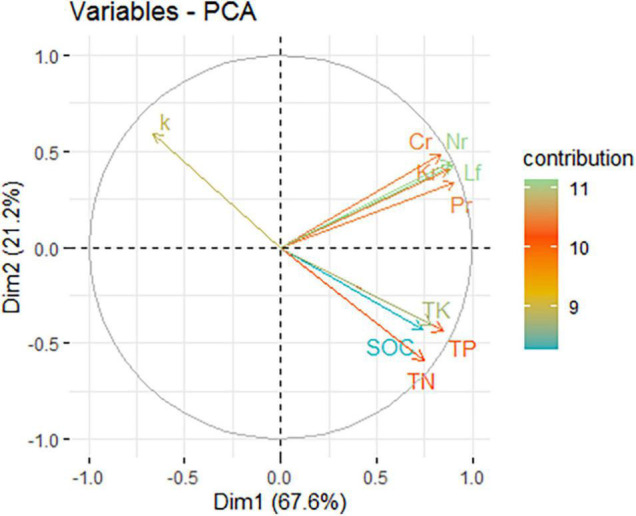
Principal component analysis of variables. The arrow line of the variables is plotted as the correlation coefficients between them and the first two principal components in the unit circle; colors indicate the contributions (%) of variables to the variance in a given principal component. SOC, soil organic carbon; TN, total nitrogen; TP, total phosphorous; TK, total potassium; Lf, annual litter yield; Cr, C return through litter in a year; Nr, N return through litter in a year; Pr, P return through litter in a year; Kr, K return through litter in a year; k, annual decay constant.

The first two principal components, component 1 (Dim.1) and component 2 (Dim.2) explained 67.6% and 21.2% of the variation, respectively (Figure 4). PCA indicated that SOC, TN, TP, and TK were negatively correlated with k. The return of C, N, P, and K was positively correlated with litter yield. This change could be attributed to changes in the stand density. Moso bamboo is characterized by its rapid growth, and before harvesting, bamboo plantations with a relatively high biomass make full use of the space and resources in soil (light, temperature, and nutrition), and resource competition is intense. Cutting reduced the density of standing bamboo and the transport of nutrients between the ramets of the clonal plants through the bamboo rhizome. This practice created an abundant, nutrient-rich environment for new trees. Therefore, a reduction in the litter decomposition rate can decrease nutrient loss and increase nutrient storage.

## Conclusion

After cutting, only New bamboo grew in SC plot, while CK and RB plot had three kinds of bamboo of different ages. However, this study clearly indicated that strip cutting did not reduce annual litter yield or nutrient return. The seasonal variation trend of litter yield in the three treatment plots was consistent, but it was found that the annual litter yield and nutrient return of N, P, and K in RB increased significantly, and PCA indicated that there was a close correlation between litter yield and nutrient return. The reabsorption efficiency of N in SC was significantly lower than in CK at the leaf changing stage. In RB, the reabsorption efficiency of P was significantly higher than in CK during shoot pregnancy and leaf changes. The difference in the reabsorption efficiency of K mainly occurred after leaf change had completed, being significantly higher in SC than in CK. Moreover, litter decomposition tests revealed that the decomposition rate of SC was significantly lower than that of CK, and the litter decomposition rate was negatively correlated with soil nutrients after a short recovery time. Based on these findings, we propose that the reduction in bamboo forest density in the strip-cutting plots provided a sufficient nutrient environment for new trees. Therefore, it is necessary to conduct a long-term study to quantify nutrient flow to explore the response of nutrient cycling to strip cutting and guide the management of bamboo forests.

## Data Availability Statement

The original contributions presented in the study are included in the article/supplementary material, further inquiries can be directed to the corresponding authors.

## Author Contributions

SF and FG designed the study and improved the English language and grammatical editing. YZ wrote the first draft of manuscript and performed the data analysis. YZ and XY did the field works. LH gave guidance and methodological advice. All authors contributed to the discussion, revision and improvement of the manuscript.

## Conflict of Interest

The authors declare that the research was conducted in the absence of any commercial or financial relationships that could be construed as a potential conflict of interest.

## Publisher’s Note

All claims expressed in this article are solely those of the authors and do not necessarily represent those of their affiliated organizations, or those of the publisher, the editors and the reviewers. Any product that may be evaluated in this article, or claim that may be made by its manufacturer, is not guaranteed or endorsed by the publisher.
